# O Tratamento com Capsinoides Promove Redução da Contratilidade dos Cardiomiócitos na Obesidade sem Modular Positivamente Parâmetros Metabólicos

**DOI:** 10.36660/abc.20250865

**Published:** 2026-07-02

**Authors:** Kiany Miranda, Késsia Cristina Carvalho Santos, Janete Corrêa Cardoso, Lucas Furtado Domingos, Fabiane Merigueti Nunes, Luisa Martins Simmer, Daniel Sesana Silva, Marina Politi Okoshi, Danilo Sales Bocalini, Ana Paula Lima-Leopoldo, André Soares Leopoldo

**Affiliations:** 1 Universidade Federal do Espírito Santo Vitória ES Brasil Universidade Federal do Espírito Santo, Vitória, ES – Brasil; 2 Universidade Estadual Paulista São Paulo SP Brasil Universidade Estadual Paulista (UNESP), São Paulo, SP – Brasil

**Keywords:** Obesidade, Dieta Hiperlipídica, Coração

## Abstract

**Fundamento:**

Os capsinoides (Cap) são compostos bioativos com propriedades termogênicas capazes de promover alterações fisiológicas e auxiliar na digestão, podendo influenciar a perda de gordura na obesidade. No entanto, seus efeitos sobre o tecido cardíaco permanecem pouco esclarecidos.

**Objetivos:**

Este estudo investigou os efeitos da administração crônica de Cap sobre a contratilidade e a morfologia cardíaca em ratos obesos (Ob) induzidos por uma dieta hiperlipídica (DHL) saturada.

**Métodos:**

Ratos Wistar machos foram distribuídos em grupos alimentados com dieta padrão (DP) ou DHL por 19 semanas. Após esse período, os animais foram redistribuídos em Controle (C), Obeso (Ob) e Obeso suplementado com capsinoides (ObCap). O grupo ObCap recebeu Cap por gavagem orogástrica (10 mg/kg). Foram realizadas avaliações nutricionais, perfil dietético, análises histológicas, comorbidades e avaliações cardíacas. O nível de significância adotado foi de 5% para todos os testes.

**Resultados:**

Os depósitos de gordura e a adiposidade total foram maiores nos animais Ob em comparação ao grupo C, sem diferenças entre Ob e ObCap. Alterações metabólicas, hormonais e lipídicas foram observadas entre Ob e ObCap. Não foram detectadas diferenças na morfometria cardíaca entre os grupos. O grupo ObCap apresentou redução na fração de encurtamento em comparação ao Ob. Quanto ao tempo de encurtamento, os animais Ob apresentaram redução em relação ao C, enquanto o grupo ObCap apresentou aumento em relação ao Ob.

**Conclusão:**

O tratamento com Cap não preveniu o acúmulo excessivo de gordura nem melhorou os parâmetros metabólicos na obesidade. Além disso, prejudicou a contratilidade dos cardiomiócitos.

**Figure:**
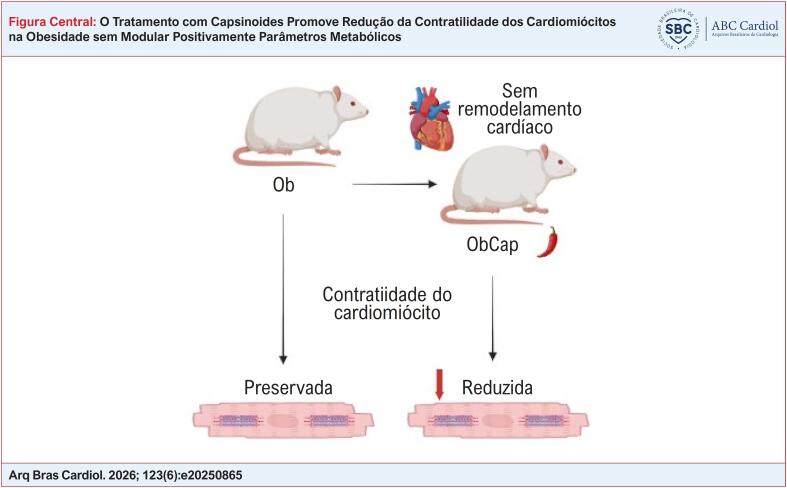
O tratamento com capsinoides compromete a função contrátil dos cardiomiócitos na presença de obesidade.

## Introdução

O sobrepeso e a obesidade são caracterizados pelo acúmulo excessivo de gordura e estão associados a diversas complicações de saúde.^1^ Em particular, a obesidade pode levar a doenças cardiovasculares, diabetes, câncer e hipertensão.^1^ Há evidências de que a obesidade está relacionada a alterações estruturais e funcionais no coração, tanto em humanos quanto em modelos animais.^2,3^ Essas alterações incluem hipertrofia dos ventrículos direito e esquerdo, aumento do átrio esquerdo e disfunção sistólica e diastólica, que podem evoluir para formas mais graves de insuficiência cardíaca.^2^

Estudos também relataram que a obesidade pode causar disfunção contrátil no coração,^3,4^ e esse dano pode ocorrer já no início da obesidade.^5^ Um aspecto interessante é o crescente uso de suplementos alimentares para fins estéticos, impulsionado principalmente pelas redes sociais.^6,7^ A busca pelo "corpo perfeito" tem estimulado a oferta desses produtos, incentivando a indústria a desenvolver cada vez mais "pílulas milagrosas", suplementos alimentares, termogênicos e compostos bioativos. Entre esses compostos bioativos, os capsinoides (Cap) têm sido sugeridos como uma estratégia não farmacológica para manter um corpo magro com baixa massa de gordura.^8,9^

Dentro da família dos Cap, o extrato seco de *Capsicum annuum* é um análogo não picante da capsaicina, derivado de pimentas doces do gênero Capsicum, pertencente à família Solanaceae. Esse extrato contém três tipos de Cap: capsiato, dihidrocapsiato e nordihidrocapsiato. Os Cap exercem efeitos semelhantes aos da capsaicina, ativando o receptor vaniloide do tipo 1 (TRPV1).^10-12^

Os efeitos antiobesidade dos Cap foram demonstrados em populações humanas, com dados epidemiológicos indicando que o consumo de alimentos contendo esses compostos pode reduzir a prevalência da obesidade.^13,14^ Alguns estudos sugerem que os Cap podem aumentar a frequência cardíaca^15,16^ e contribuir para a prevenção de doenças cardiovasculares.^17,18^ Além disso, há evidências de que esses compostos exercem efeitos benéficos sobre o sistema cardiovascular, ajudando a prevenir condições como hipertensão e aterosclerose.^19,20^

Esses achados ressaltam a necessidade de uma avaliação mais abrangente das respostas metabólicas e cardíacas à administração crônica de Cap e suas implicações para estratégias terapêuticas no contexto da obesidade. Assim, o presente estudo foi desenvolvido para testar a hipótese de que a administração crônica de Cap modula positivamente a contratilidade dos cardiomiócitos e os parâmetros metabólicos.

## Materiais e métodos

### Cuidados com os animais e delineamento experimental

Todos os procedimentos experimentais foram aprovados pelo Comitê de Ética no Uso de Animais (CEUA) da UFES, sob o protocolo nº 08/2022. Ratos Wistar (n = 41), com 30 dias de idade, foram mantidos em ambiente controlado, com ciclo claro/escuro de 12 horas, umidade relativa de 55 ± 5% e temperatura de 24 ± 2 °C. Um grupo recebeu dieta padrão (DP, n = 16; AIN-93, fornecendo 9,4% das calorias provenientes de gordura e densidade energética de 3,81 kcal/g), enquanto o outro grupo foi alimentado com dieta hiperlipídica (DHL, n = 25; fornecendo 45,3% das calorias provenientes de gordura e densidade energética de 4,82 kcal/g) (Pragsoluções Biociências^®^, Brasil). A composição específica das dietas é apresentada na Tabela 1. Durante todo o estudo, os animais tiveram acesso irrestrito à água purificada e à ração (40 g/dia).

**Tabela 1:** Composição e valores de macronutrientes das dietas

Componentes (g/kg)	Dietas	
	Dieta padrão (AIN - 93)	Dieta hiperlipidica
Amido de milho	465	271
Caseína	140	170
Amido dextrinizado	155	155
Sacarose	100	60
Óleo de soja	40	28
Celulose	50	50
L-Cisteína	1,8	3,5
Mineral mix*	35	35
Vitamina mix*	10	10
Bitartarato de colina	2,5	2,5
Banha	-	21,5
Hidroxitolueno butilado	0,016	-
**Total**	**1000**	**1000**
**Macronutrientes (%)**		
Proteína	14,7	14,1
Carboidrato	75,8	40,4
Lipídios	9,47	45,4

Mistura de vitaminas e minerais: vitamina A, vitamina D_3_*, vitaina K*_3_*, complexo B, ácido pantotênico, ácido fólico, biotina, colina, vit. C, selênio, ferro, cobre, manganês, iodo, zinco, cobalto, cálcio e fósforo.*

**Figura 1:**
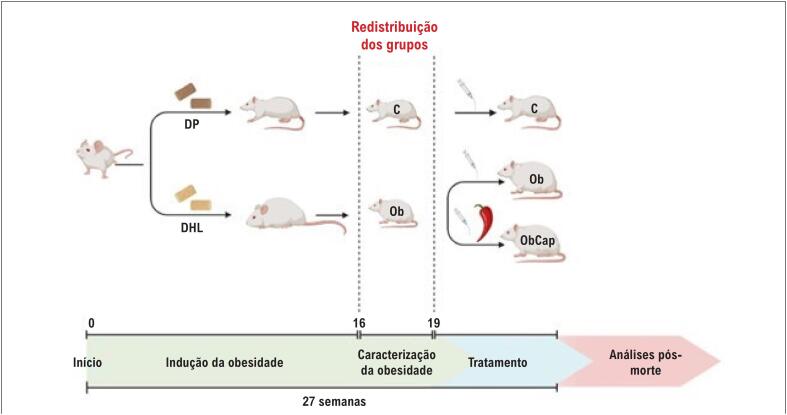
Representação esquemática do protocolo experimental (27 semanas). DP: dieta padrão (n = 16); DHL: dieta hiperlipídica (n = 25); Controle (C, n = 10), Obeso (Ob, n = 12) e Obeso tratado com capsinoides (ObCap, n = 13).

O protocolo experimental (esquematizado na Figura 1) teve duração total de 27 semanas, dividido em duas fases: (i) uma fase de indução e manutenção da obesidade, com duração de 19 semanas, seguida por (ii) um período de 8 semanas de tratamento com Cap. Ao final da 19ª semana, os ratos foram estratificados de acordo com o peso corporal, distribuídos aleatoriamente e alocados em três grupos com base na presença ou ausência de tratamento com Cap: Controle (C, n = 10), Obeso (Ob, n = 12) e Obeso tratado com Cap (ObCap, n = 13).

### Administração de Capsinoides

O grupo ObCap recebeu Cap (CAP; Infinity Pharma, Brasil) diariamente por gavagem orogástrica, na dosagem de 10 mg de Cap por kg de peso corporal, diluídos em 1 ml de água por kg de peso corporal, durante um período de 8 semanas.^21^ A dose de Cap foi recalculada semanalmente para acompanhar as variações de peso corporal, garantindo administração consistente ao longo do estudo. Os grupos C e Ob receberam gavagem com veículo em volumes equivalentes.

### Avaliação nutricional

A avaliação nutricional foi realizada por meio de medidas semanais de peso corporal, da soma da gordura epididimal e visceral, e do cálculo do índice de adiposidade (gordura total/peso final × 100).^22,23^

### Perfis bioquímico e hormonal

Os perfis glicêmicos dos animais foram avaliados após jejum de 6 horas na última semana do protocolo. Os níveis glicêmicos foram medidos no basal e aos 30, 60, 90 e 120 minutos após injeção intraperitoneal de solução de glicose a 50%. A tolerância à glicose foi avaliada pelo cálculo da área sob a curva (AUC) da glicose. A resistência à insulina foi analisada pelo índice HOMA-IR (*homeostatic model assessment*), calculado como [concentração de insulina em jejum (μU/mL) × glicemia em jejum (mmol/L)/22,5], com base nas concentrações plasmáticas de glicose e insulina em jejum.

O perfil hormonal (leptina e insulina) foi determinado com kits específicos (Anti-Insulin Merck^®^ EZRMI-13K). O perfil lipídico foi avaliado pela dosagem de triglicerídeos (TG) e colesterol total plasmático utilizando kits específicos (Bioclin Bioquímica, Brasil).

### Avaliação do remodelamento cardíaco – Análise macroscópica

O remodelamento macroscópico, indicativo de hipertrofia miocárdica, foi avaliado pela massa cardíaca total e normalizado pelo comprimento da tíbia.

### Análise histológica

A área de secção transversal do ventrículo esquerdo dos animais de cada grupo foi fixada em paraformaldeído a 4% e incluída em parafina. Foram obtidos cortes de 6 μm, corados com hematoxilina-eosina (HE) para determinação da área de secção transversal dos miócitos. Todas as análises foram realizadas de forma cega.

### Isolamento de cardiomiócitos

A análise funcional cardíaca *in vitro* foi realizada utilizando a técnica de isolamento de cardiomiócitos.^18^ A contratilidade celular foi avaliada pela técnica de variação do comprimento dos cardiomiócitos, com auxílio de microscópio invertido equipado com sistema de detecção de borda. As células, mantidas em solução de Tyrode e estimuladas a 1 Hz, tiveram suas respostas contráteis registradas e analisadas quanto a fração de encurtamento (%), taxas máximas de encurtamento e relaxamento, e tempo para 50% do pico de encurtamento e relaxamento.^21^ Todas as análises foram realizadas de forma cega.

### Análise estatística

A distribuição dos dados foi avaliada pelo teste de normalidade de Shapiro-Wilk. Os resultados são apresentados como média ± desvio padrão (DP). As comparações entre os grupos DP e DHL foram realizadas pelo teste t de Student para amostras independentes. Para comparação entre os grupos C, Ob e ObCap foi utilizada ANOVA de uma via, seguida do teste *post hoc* de Tukey. Para avaliar alterações no peso corporal durante o período de exposição às dietas experimentais, foi utilizada ANOVA de duas vias, seguida do teste *post hoc* de Bonferroni. Durante o período de tratamento com Cap, a massa corporal e os testes de tolerância à glicose (GTT) foram avaliados por ANOVA de duas vias, com análise subsequente pelo teste *post hoc* de Bonferroni. Para avaliação dos parâmetros de contratilidade, foi utilizado o teste de Kruskal-Wallis, seguido do teste *post hoc* de Dunn. O nível de significância adotado para todos os testes foi de 5%. As análises estatísticas e representações gráficas foram realizadas utilizando o software GraphPad Prism 9.0.

## Resultados

### Caracterização da obesidade

**Figura 2:**
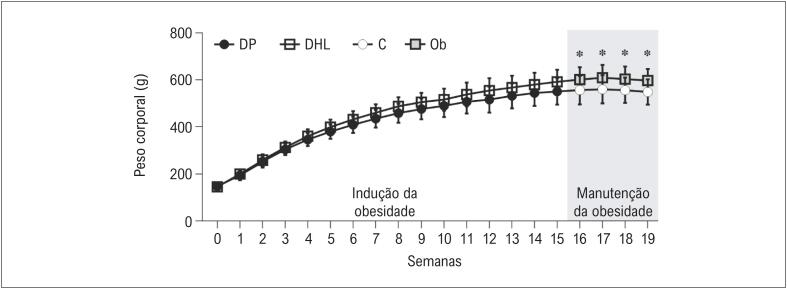
Evolução do peso corporal no grupo alimentado com dieta padrão (DP, n = 16), submetido a uma dieta normocalórica, e no grupo alimentado com dieta hiperlipídica (DHL, n = 25) durante o período de indução da obesidade. Após o início e a caracterização da obesidade, os grupos foram reclassificados como Controle (C, n = 16) e Obeso (Ob, n = 25). Os valores são expressos como média ± desvio padrão. ANOVA de duas vias seguida do teste *post hoc* de Bonferroni. Os dados foram considerados significativos quando p < 0.05 para Ob vs. C.

Inicialmente, os animais apresentaram pesos corporais semelhantes durante as primeiras 15 semanas do protocolo experimental (Figura 2). A partir da 16ª semana, entretanto, o grupo DHL (465 ± 143 g) apresentou peso corporal significativamente maior em comparação ao grupo DP (438 ± 128 g), marcando o início da obesidade. O período entre a 16ª e a 19ª semanas foi designado como fase de manutenção da obesidade, durante a qual os grupos foram reclassificados como Controle (C) e Obesidade (Ob).

### Administração crônica de capsinoides

Considerando o perfil nutricional, durante a administração crônica de Cap foi observada uma redução significativa na ingestão alimentar no grupo Ob (15,2 ± 1,7 g/dia) em comparação ao grupo C (17,6 ± 1,5 g/dia). No entanto, o consumo calórico e a eficiência alimentar não diferiram significativamente entre os grupos (dados não mostrados). Assim, o tratamento com Cap não promoveu melhorias no perfil nutricional em condições de obesidade.

A Tabela 2 apresenta os fatores de risco cardiometabólicos com base nos parâmetros biométricos dos grupos após 8 semanas de administração crônica de Cap. Em relação ao peso corporal, o peso corporal inicial (PCI) do grupo Ob foi maior em comparação ao grupo C.

**Tabela 2:** Perfil nutricional e composição corporal após o tratamento com Cap

Variáveis	Grupos		
	C (n=10)	Ob (n=12)	ObCap (n=13)
Ingestão alimentar (g/dia)	17,6 ± 1,5	15,2 ± 1,7*	16,5 ± 7,3
Consumo calórico (Kcal/day)	67 ± 6	73 ± 8	70 ± 10
Eficiência alimentar (%)	0,3 ± 1,4	0,7 ± 1,2	0,4 ± 0,9
Peso corporal inicial (g)	539 ± 50	593 ± 53*	600 ± 48
Peso corporal final (g)	541 ± 45	583 ± 54	608 ± 44
Gordura epididimal (g)	8,3 ± 1,9	1,6 ± 1,9**	13,0 ± 2,2
Gordura visceral (g)	7,0 ± 1,6	11,7 ± 2,4****	13,0 ± 2,7
Soma dos depósitos de gordura (g)	28,6 ± 3,5	43,6 ± 8,1**	52,4 ± 12,8
Índice de adiposidade (%)	5,3 ± 0,8	7,5 ± 1,1**	8,6 ± 1,9

Os dados são apresentados como média ± DP. Controle (C), Obeso (Ob) e Obeso tratado com capsinoides (ObCap). ANOVA de uma via seguida do teste post hoc de Tukey.

* p < 0,05;

** p < 0,01; *p < 0,001;

**** – vs. C.

Como esperado, os ratos do grupo Ob apresentaram valores elevados para todos os parâmetros de adiposidade em comparação ao grupo C, confirmando a eficácia da DHL na indução da obesidade. Entretanto, o tratamento com Cap não reduziu nem preveniu o acúmulo de gordura associado à obesidade (Tabela 2).

**Figura 3:**
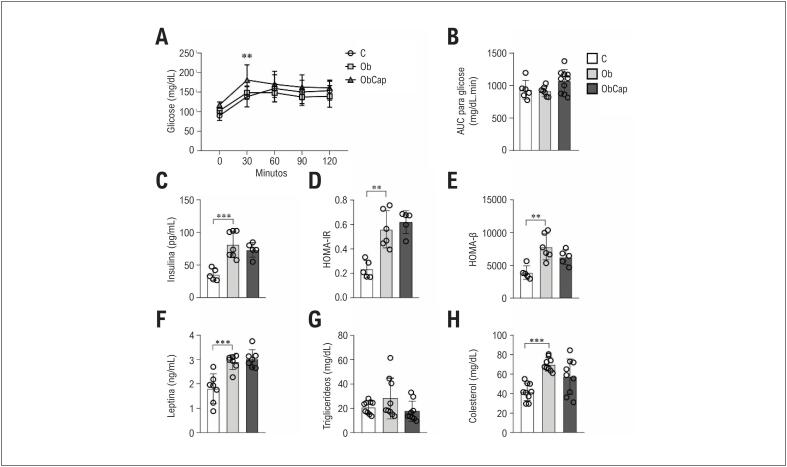
Perfil lipídico, glicêmico e hormonal após o tratamento com Cap. A) Teste de Tolerância à Glicose (GTT), **p < 0,01 ObCap vs. Ob; Controle (C, n = 6), Obeso (Ob, n = 7) e Obeso tratado com capsinoides (ObCap, n = 10). B) Área sob a curva (AUC) da glicose; Controle (C, n = 6), Obeso (Ob, n = 7) e Obeso tratado com capsinoides (ObCap, n = 10). C) Insulina; Controle (C, n = 5), Obeso (Ob, n = 7) e Obeso tratado com capsinoides (ObCap, n = 5). D) HOMA-IR (Homeostatic Model Assessment – Insulin Resistance); Controle (C, n = 5), Obeso (Ob, n = 6) e Obeso tratado com capsinoides (ObCap, n = 5). E) HOMA-β (Homeostatic Model Assessment – Beta); Controle (C, n = 5), Obeso (Ob, n = 6) e Obeso tratado com capsinoides (ObCap, n = 5). F) Leptina; Controle (C, n = 7), Obeso (Ob, n = 7) e Obeso tratado com capsinoides (ObCap, n = 7). G) Triglicerídeos; Controle (C, n = 10), Obeso (Ob, n = 10) e Obeso tratado com capsinoides (ObCap, n = 10). H) Colesterol; Controle (C, n = 9), Obeso (Ob, n = 9) e Obeso tratado com capsinoides (ObCap, n = 9). Os valores são expressos como média ± DP e valores individuais. As comparações estatísticas foram realizadas por ANOVA de duas vias seguida do teste de Bonferroni para comparações múltiplas (A) ou por ANOVA de uma via seguida do teste de Tukey para comparações múltiplas (B–H). Os dados foram considerados significativos quando p < 0,05; p < 0,01; p < 0,001.

A Figura 3 ilustra as comorbidades relacionadas à obesidade nos grupos após o tratamento crônico com Cap. No basal, o grupo ObCap apresentou níveis glicêmicos elevados em comparação ao grupo Ob. Não foram observadas diferenças significativas nos demais pontos de avaliação (Figura 3A). Além disso, não houve diferenças na área sob a curva (AUC) da glicose ou nos níveis de TG entre os grupos (Figuras 3B e 3G).

Os níveis de insulina foram significativamente elevados no grupo Ob em comparação ao grupo C, mas não houve diferença significativa entre os grupos Ob e ObCap (Figura 3C). Um padrão semelhante foi observado para o índice HOMA-IR, com o grupo Ob apresentando valores mais altos em relação ao grupo C. Para o índice HOMA-β, o grupo Ob mostrou aumento em comparação ao grupo C (Figuras 3D e 3E). É importante destacar que não houve diferenças nesses índices entre Ob e ObCap, sugerindo ausência de efeito do tratamento com Cap.

Em relação aos níveis de leptina, o grupo Ob apresentou aumento significativo em comparação ao grupo C, sem diferença significativa entre os grupos Ob e ObCap (Figura 3F). O grupo Ob também apresentou níveis mais elevados de colesterol em comparação ao grupo C (Figura 3H).

**Figura 4:**
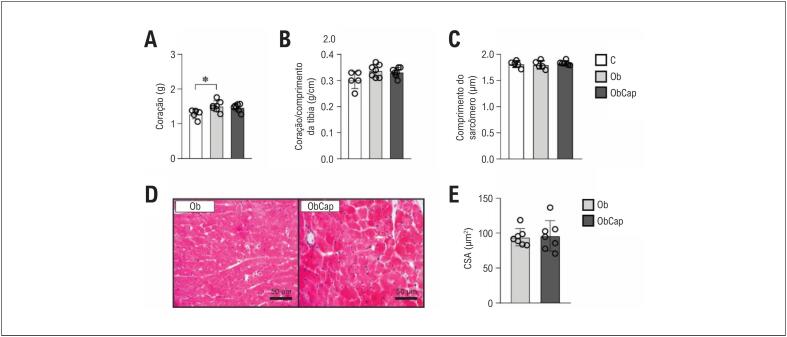
Características morfológicas cardíacas após o tratamento com Cap. A) Peso do coração; Controle (C, n = 5), Obeso (Ob, n = 7) e Obeso tratado com capsinoides (ObCap, n = 7). B) Relação coração/comprimento da tíbia; Controle (C, n = 5), Obeso (Ob, n = 7) e Obeso tratado com capsinoides (ObCap, n = 7). C) Comprimento do sarcômero; Controle (C, n = 5), Obeso (Ob, n = 5) e Obeso tratado com capsinoides (ObCap, n = 5). D) Imagens representativas de cortes transversais do ventrículo esquerdo (×40) corados com H&E; barra de escala = 50 µm. E) Gráfico de barras da área seccional transversa (CSA); Obeso (Ob, n = 7) e Obeso tratado com capsinoides (ObCap, n = 7). Os valores são expressos como média ± DP e valores individuais. As comparações estatísticas foram realizadas por ANOVA de uma via seguida do teste de Tukey para comparações múltiplas (A–C, E). Os dados foram considerados significativos quando p < 0,05 vs. C.

Quanto à morfologia cardíaca, os resultados mostraram que o grupo Ob apresentou aumento no peso do coração em relação ao grupo C (Figura 4A); entretanto, quando o peso do coração foi normalizado pelo comprimento da tíbia, não houve diferenças significativas entre os grupos (Figura 4B). A morfometria celular foi avaliada por meio do comprimento do sarcômero e da área seccional transversa dos cardiomiócitos. Nem o tratamento com Cap nem a obesidade foram capazes de induzir alterações nesses parâmetros (Figuras 4C-E).

**Figura 5:**
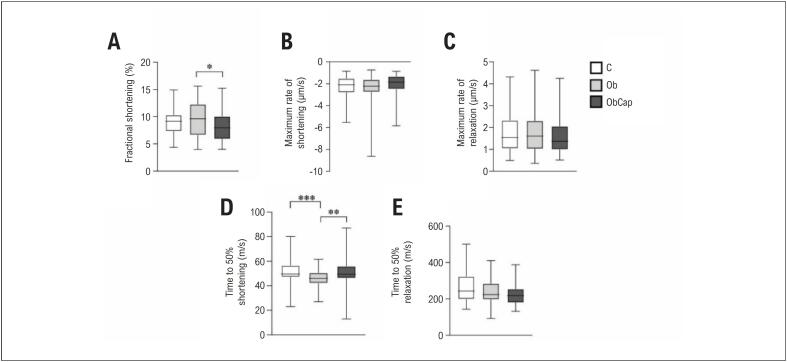
Efeito dos capsinoides sobre os parâmetros funcionais de cardiomiócitos isolados. A) Encurtamento fracionado expresso como % do comprimento celular em repouso; Controle (C, n = 65 células), Obeso (Ob, n = 61 células) e Obeso tratado com capsinoides (ObCap, n = 60 células). B) Taxa máxima de encurtamento; Controle (C, n = 65 células), Obeso (Ob, n = 61 células) e Obeso tratado com capsinoides (ObCap, n = 60 células). C) Taxa máxima de relaxamento; Controle (C, n = 65 células), Obeso (Ob, n = 60 células) e Obeso tratado com capsinoides (ObCap, n = 60 células). D) Tempo para 50% do encurtamento; Controle (C, n = 64 células), Obeso (Ob, n = 60 células) e Obeso tratado com capsinoides (ObCap, n = 60 células). E) Tempo para 50% da relaxação; Controle (C, n = 65 células), Obeso (Ob, n = 61 células) e Obeso tratado com capsinoides (ObCap, n = 59 células). Os dados são expressos como mediana ± intervalos interquartis. As comparações estatísticas foram realizadas utilizando o teste de Kruskal-Wallis seguido do teste de Dunn para comparações múltiplas (A–E). Os dados foram considerados significativos quando p < 0,05; p < 0,01; p < 0,001.

Para melhor compreender os efeitos celulares do tratamento com Cap na presença de obesidade, foram realizados experimentos com cardiomiócitos isolados para análise da função contrátil. Surpreendentemente, o tratamento com Cap promoveu uma modulação significativa da contratilidade dos cardiomiócitos, evidenciada pela redução da fração de encurtamento (%) no grupo ObCap em comparação ao grupo Ob (Figura 5A). Parâmetros como as taxas máximas de encurtamento e relaxamento permaneceram inalterados nos grupos Ob e ObCap (Figuras 5B-C). A obesidade reduziu o tempo para 50% do encurtamento em comparação ao grupo C, enquanto o grupo ObCap apresentou aumento nesse parâmetro em relação ao grupo Ob (Figura 5D). O tempo para 50% do relaxamento não apresentou diferenças entre os grupos (Figura 5E).

## Discussão

Os principais resultados demonstraram que a administração crônica de Cap, como abordagem farmacológica para o tratamento da obesidade, não foi eficaz na redução da adiposidade. Além disso, a administração crônica de Cap não melhorou os perfis glicêmico, lipídico ou hormonal e não conseguiu prevenir a hipercolesterolemia isolada e a hiperinsulinemia associadas à obesidade. No entanto, especificamente no coração, o tratamento com Cap comprometeu a contratilidade celular, evidenciada pela redução do encurtamento fracionado e pelo aumento do tempo para 50% de encurtamento, destacando sua capacidade de modular a contratilidade dos cardiomiócitos.

Diversas intervenções farmacológicas têm sido empregadas para tratar a obesidade^24,25^ e, entre essas abordagens, Cap tem sido investigado como uma ferramenta não farmacológica com efeitos promissores na redução da massa corporal e da adiposidade em camundongos obesos.^26,27^

Os resultados indicam que, a partir da 16ª semana, os grupos Ob apresentaram diferenças estatisticamente significativas no peso corporal, mantendo essa diferença até o final do protocolo de indução e manutenção da obesidade (19ª semana). No contexto do presente estudo, os dados mostram que a intervenção com Cap não foi eficaz na redução dos parâmetros corporais ou da adiposidade. De modo geral, nossos resultados demonstram que o tratamento crônico com Cap não teve impacto no acúmulo de gordura corporal, revelando uma interação até então pouco explorada. Esse achado é relevante porque Cap tem emergido como um composto com potencial para o controle e a redução da obesidade, dada sua capacidade relatada de aumentar a expressão de proteínas relacionadas ao metabolismo lipídico, elevar o consumo de oxigênio e reduzir a massa corporal e os depósitos de gordura.^28^

Uma possível explicação para a ausência de efeito de Cap sobre o peso corporal e a adiposidade pode estar relacionada à dose utilizada. Além disso, diferenças metodológicas — como variações na composição da dieta (com ou sem banha) e diferenças na duração do tratamento — podem gerar efeitos de curto prazo que não se mantêm no longo prazo.^24,29,30^ Outro ponto relevante é que alguns estudos que relataram resultados positivos com capsaicina ou Capsicum annuum administraram o composto misturado diretamente à ração, o que pode influenciar a quantidade total ingerida por cada animal.^25,30^

Embora a literatura disponível sobre o mecanismo de ação de Cap seja limitada, este estudo não avaliou diretamente a expressão, atividade ou modulação de possíveis alvos mecanísticos. No entanto, evidências relacionadas aos seus análogos são relevantes, pois alguns desses compostos podem produzir efeitos semelhantes aos capsaicinóides, como promover termogênese, estimular o metabolismo energético e lipídico e reduzir a massa corporal e o tecido adiposo.^17,18^ Além disso, esses compostos podem melhorar a tolerância à glicose, apresentar atividade anti-hiperlipidêmica e demonstrar propriedades anti-inflamatórias e antioxidantes.^8,21^

A capsaicina se destaca nesse contexto porque ativa o TRPV1 e pode exercer efeitos benéficos na obesidade.^20^ Esses mecanismos foram considerados na discussão como possíveis explicações para os efeitos observados. No entanto, permanecem lacunas importantes quanto à ação específica de Cap e à possibilidade de que seus efeitos sejam semelhantes aos descritos para a capsaicina. Portanto, estudos adicionais e direcionados são necessários para investigar o papel de Cap e os mecanismos envolvidos em sua ação.

Nesse contexto, o estudo de Kang et al.,^20^ que utilizou um modelo de obesidade induzida por DHL em camundongos, demonstrou que a capsaicina dietética pode reduzir a intolerância à glicose induzida pela obesidade *in vivo*. Além disso, os benefícios da capsaicina estão associados à redução de fenótipos inflamatórios no tecido adiposo e no fígado, dois tecidos periféricos que desempenham papéis cruciais na resposta à insulina. Esses achados sugerem que a capsaicina dietética pode combater o desenvolvimento de intolerância à glicose associada à obesidade e, possivelmente, a resistência à insulina. De acordo com Kwon et al.,^26^ esse efeito pode ser atribuído à ativação do TRPV1 no pâncreas, que pode induzir a liberação do peptídeo relacionado ao gene da calcitonina (CGRP) e estimular a secreção de insulina. Além disso, agonistas de TRPV1 podem aumentar os níveis intracelulares de Ca^2+^ em células β, expandir a massa dessas células e promover maior secreção de insulina. No entanto, para compreender melhor os mecanismos que levam a esses efeitos, são necessários estudos futuros que investiguem especificamente a ação dos Cap.

Em contraste com essa ideia, nossos dados mostram claramente que a administração crônica de Cap não conseguiu prevenir a hiperinsulinemia e a resistência à insulina induzidas pela obesidade. Nossos achados são corroborados por evidências anteriores demonstrando que o tratamento com Cap não melhorou os níveis de insulina, a resistência à insulina ou as concentrações de leptina em ratos obesos.^21^

Da mesma forma, nossos achados mostram que o tratamento com Cap não foi eficaz em reverter a hipercolesterolemia isolada. Nesse contexto, Cansian^8^ também investigou os efeitos do extrato seco de Capsicum annuum L. (40%) sobre o perfil lipídico de ratos obesos tratados por 6 semanas. No entanto, não foram observadas alterações nos níveis séricos de colesterol total, HDL e TG. Por outro lado, um estudo com capsiato e dihidrocapsiato sintetizados enzimaticamente encontrou que o tratamento resultou em redução dos níveis séricos de colesterol e lipídios.^31^

A associação entre dieta e aumento da gordura corporal tem sido relacionada ao surgimento de diversas comorbidades, elevando o risco de desenvolvimento de doenças cardiovasculares.^32^ A literatura frequentemente relata que a obesidade promove remodelamento cardíaco, caracterizado por alterações morfológicas, moleculares e funcionais decorrentes de mudanças no volume, massa e função cardíaca em resposta a agressões fisiopatológicas.^2,33^

Nossos resultados não mostraram alterações na morfologia cardíaca entre os diferentes grupos do experimento. O aumento do peso absoluto do coração observado nos animais obesos não persistiu após a normalização pelo comprimento da tíbia, indicando que o crescimento cardíaco ocorreu de forma proporcional ao aumento do peso corporal. Além disso, as análises não mostraram aumento da área de secção transversal (CSA) ou do comprimento do sarcômero, sugerindo que as alterações observadas refletem uma adaptação fisiológica ao aumento da massa corporal, sem evidências de hipertrofia cardíaca patológica.

No presente estudo, demonstramos pela primeira vez que o tratamento crônico com Cap reduziu a contratilidade dos cardiomiócitos na obesidade. Essa descoberta é importante, pois há pouca informação disponível sobre os efeitos de Cap no coração e na contratilidade celular no contexto da obesidade. Estudos que investigam a função contrátil cardíaca em modelos experimentais de obesidade revelam deterioração da capacidade de contração, evidenciada pela diminuição do percentual e da velocidade de encurtamento dos cardiomiócitos.^24,34^

Nossos resultados mostram que a fração de encurtamento dos cardiomiócitos de animais obesos está preservada, com redução no tempo necessário para atingir 50% do encurtamento máximo. Esse achado pode estar associado a alterações no manejo intracelular de cálcio ou a mudanças na sensibilidade das proteínas contráteis; no entanto, os mecanismos subjacentes ainda não foram avaliados diretamente. Portanto, apesar da preservação da função contrátil, a alteração na velocidade de contração sugere modificações na mecânica dos cardiomiócitos relacionadas à obesidade. Embora a literatura descreva consistentemente prejuízos na contratilidade miocárdica associados à obesidade, nossos resultados não demonstram tal disfunção. Essa discrepância pode ser explicada por variações no modelo experimental, como espécie, linhagem ou protocolo dietético, que podem influenciar a suscetibilidade dos cardiomiócitos às alterações funcionais.^33^ Também é possível que ocorram adaptações celulares, como aumento da sensibilidade das miofibrilas ao cálcio.^35^

Quando comparamos os cardiomiócitos de animais obesos tratados com Cap com aqueles do grupo obeso não tratado, observamos uma redução na fração de encurtamento e um aumento no tempo necessário para atingir 50% do encurtamento máximo (ver ilustração central). Esses resultados indicam prejuízo da função contrátil celular tanto em magnitude quanto em velocidade. Enquanto o grupo obeso manteve a amplitude de contração, o tratamento com Cap reduziu a capacidade de encurtamento e desacelerou a contração, sugerindo menor eficiência mecânica. Esse efeito pode estar relacionado a alterações no manejo intracelular de cálcio, possivelmente por meio da modulação de proteínas reguladoras como SERCA2a e RYR2, o que poderia reduzir a disponibilidade de cálcio para a contração.^35^ Estudos adicionais são necessários para elucidar os mecanismos envolvidos na ação de Cap no sistema cardíaco. Uma limitação deste estudo é a ausência de avaliação da função cardíaca in vivo, o que restringe nossa compreensão sobre até que ponto as alterações observadas em nível celular se refletem na função do órgão.

Embora não exista literatura descrevendo o mecanismo pelo qual o tratamento com Cap afeta a contratilidade dos cardiomiócitos, evidências sobre seu análogo, a capsaicina, fornecem um contexto relevante. A capsaicina é conhecida por atuar no sistema cardíaco por meio do receptor TRPV1, exercendo efeitos cardioprotetores ao reduzir o tamanho do infarto e estimular a vasodilatação.^36^ No entanto, permanecem lacunas significativas na compreensão de como Cap atua no sistema cardíaco e se seus efeitos se assemelham aos atribuídos à capsaicina. Portanto, estudos adicionais são necessários para investigar o papel de Cap na função e na contratilidade cardíaca, bem como os mecanismos envolvidos.

Nesse contexto, o tratamento com capsaicina demonstrou melhorar o controle autonômico cardiovascular em ratos recém-nascidos, restaurando a frequência cardíaca. Em doses elevadas, a capsaicina também foi capaz de aumentar a modulação simpática do tônus vascular.^37^ Estudos demonstraram que o efeito agudo da capsaicina em culturas de cardiomiócitos aumenta a concentração intracelular de Ca^2+^ e atenua o encurtamento fracionado por meio da estimulação do TRPV1, evidenciado pelo uso de um bloqueador específico de TRPV1 que impediu o aumento de Ca^2+^ intracelular induzido pela capsaicina.^38,39^

Mais recentemente, Isaev et al.^40^ investigaram os efeitos da capsaicina sobre correntes iônicas dependentes de voltagem em cardiomiócitos ventriculares de coelho. A capsaicina inibiu as correntes de K+ ativadas rapidamente (IKr) e lentamente (IKs), bem como a corrente transitória de saída de K+ (Ito). Em concentrações mais altas, a capsaicina também suprimiu as correntes de Na^+^ e Ca^2+^ dependentes de voltagem e a corrente retificadora interna IK1. No conjunto, esses achados sugerem que a capsaicina influencia a eletrofisiologia cardíaca ao interagir com múltiplos canais iônicos. Portanto, recomenda-se cautela ao administrar capsaicina a indivíduos com canalopatias cardíacas ou predisposição a arritmias, como aqueles com doença cardíaca isquêmica.

Em conjunto, nossos resultados fornecem evidências de que Cap induz prejuízo na contratilidade dos cardiomiócitos no contexto da obesidade. Esses achados sugerem que, apesar da preservação morfológica, o tratamento com Cap pode afetar negativamente a mecânica celular dos cardiomiócitos.

## Conclusão

O tratamento com Cap modula a contratilidade dos cardiomiócitos na presença de obesidade. Nossos achados fornecem novos *insights* sobre a relação entre o tratamento com Cap e a contração dos cardiomiócitos, mas seu potencial terapêutico ainda precisa ser mais bem esclarecido. Além disso, o tratamento com Cap não melhorou o fenótipo de acúmulo de gordura causado pela indução da obesidade, nem promoveu melhorias nos parâmetros metabólicos.

## Data Availability

Os conteúdos subjacentes ao texto da pesquisa estão contidos no manuscrito.
