# Supplementary material for: O Tratamento com Capsinoides Promove Redução da Contratilidade dos Cardiomiócitos na Obesidade sem Modular Positivamente Parâmetros Metabólicos
Source: Arq Bras Cardiol. 2026 Jul 2;123(6):e20250865. [Article in Portuguese] doi: 10.36660/abc.20250865 (PMC13399221; doi:10.36660/abc.20250865)
Supplement: Translation

# Translation: Treatment with Capsinoids Promotes Impaired Cardiomyocyte Contractility in Obesity without Positively Modulating Metabolic Parameters

**DOI:** 10.36660/abc.20250865i

**Authors:** Kiany Miranda, Késsia Cristina Carvalho Santos, Janete Corrêa Cardoso, Lucas Furtado Domingos, Fabiane Merigueti Nunes, Luisa Martins Simmer, Daniel Sesana Silva, Marina Politi Okoshi, Danilo Sales Bocalini, Ana Paula Lima-Leopoldo, André Soares Leopoldo

**Affiliations:** 1 Vitória ES Brazil Universidade Federal do Espírito Santo, Vitória, ES – Brazil; 2 São Paulo SP Brazil Universidade Estadual Paulista (UNESP), São Paulo, SP – Brazil

**Keywords:** Obesity, High-Fat Diet, Heart

## Abstract

**Background:**

Capsinoids (Cap) are bioactive compounds with thermogenic properties capable of promoting physiological changes and aiding digestion, potentially influencing fat loss in obesity. However, their effects on cardiac tissue remain unclear.

**Objective:**

This study investigated the effects of chronic Cap administration on cardiac contractility and morphology in obese (Ob) rats induced by a saturated high-fat diet (HFD).

**Methods:**

Male Wistar rats were assigned to standard diet (SD) or HFD groups for 19 weeks. After this period, animals were redistributed into Control (C), Obese (Ob), and Obese supplemented with capsinoids (ObCap). The ObCap group received Cap via orogastric gavage (10 mg/kg). Nutritional assessments, dietary profile, histological analyses, comorbidities, and cardiac evaluations were performed. A significance level of 5% was adopted for all tests.

**Results:**

Fat deposits and total adiposity were higher in Ob animals compared with the C group, with no differences between Ob and ObCap. Metabolic, hormonal, and lipid alterations were observed between Ob and ObCap. No differences in cardiac morphometry were detected among groups. The ObCap group exhibited reduced fractional shortening compared with Ob. Regarding shortening time, Ob animals showed a decrease compared with C, whereas ObCap showed an increase compared with Ob.

**Conclusion:**

Treatment with Cap did not prevent excessive fat accumulation nor improve metabolic parameters in obesity. Moreover, it impaired cardiomyocyte contractility.

## Introduction

Overweight and obesity are characterized by excessive fat accumulation and are associated with several health complications.^1^ In particular, obesity can lead to cardiovascular disease, diabetes, cancer, and hypertension.^1^ There is evidence that obesity is linked to structural and functional changes in the heart in both humans and animal models.^2,3^ These changes include hypertrophy of the right and left ventricles, enlargement of the left atrium, and systolic and diastolic dysfunction, which may progress to more severe forms of heart failure.^2^

Studies have also reported that obesity can cause contractile dysfunction in the heart,^3,4^ and this damage may occur as early as the onset of obesity.^5^ An interesting aspect is the increasing use of dietary supplements for aesthetic purposes, largely driven by social networks.^6,7^ The pursuit of the "perfect" body has fueled the supply of such products, encouraging the industry to develop more and more "miracle pills," dietary supplements, thermogenics, and bioactive compounds. Among these bioactive compounds, capsinoids (Cap) have been suggested as a non-pharmacological strategy to maintain a lean body with low fat mass.^8,9^

Within the Cap family, *Capsicum annuum* dry extract is a non-pungent analog of capsaicin derived from sweet peppers of the genus Capsicum in the family Solanaceae. This extract contains three types of Cap: capsiate, dihydrocapsiate, and nordihydrocapsiate. Cap exert effects similar to capsaicin by activating transient receptor potential vanilloid type 1 (TRPV1).^10-12^

The anti-obesity effects of Cap have been demonstrated in human populations, with epidemiological data indicating that consumption of foods containing these compounds may reduce the prevalence of obesity.^13,14^ Some studies suggest that Cap can increase heart rate^15,16^ and may contribute to the prevention of cardiovascular disease.^17,18^ Furthermore, there is evidence that these compounds exert beneficial effects on the cardiovascular system, helping to prevent conditions such as hypertension and atherosclerosis.^19,20^

These findings underscore the need for a more comprehensive evaluation of the metabolic and cardiac responses to chronic Cap administration and their implications for therapeutic strategies in the context of obesity. Accordingly, the present study was designed to test the hypothesis that chronic administration of Cap positively modulates cardiomyocyte contractility and metabolic parameters.

**Figure:** Treatment with capsinoids compromises the contractile function of cardiomyocytes in the presence of obesity.

## Materials and methods

### Animal care and experimental design

All experimental procedures were approved by the Ethics Committee on the Use of Animals (CEUA) of UFES under protocol number 08/2022. Wistar rats (n = 41), 30 days old, were housed in a controlled environment with a 12-hour light/dark cycle, relative humidity of 55 ± 5%, and temperature of 24 ± 2 °C. One group received a standard diet (SD, n = 16; AIN-93, providing 9.4% of calories from fat and an energy density of 3.81 kcal/g), while the other group was fed a high-fat diet (HFD, n = 25; providing 45.3% of calories from fat and an energy density of 4.82 kcal/g) (Pragsoluções Biociências^®^, Brazil). The specific composition of the diets is presented in Table 1. Throughout the study, all rats had unrestricted access to purified water and feed (40 g/day).

**Table 1:** Composition and macronutrient values of diets

Components (g/kg)	Diets	
	SD (AIN - 93)	HFD
Corn starch	465	271
Casein	140	170
Dextrinized starch	155	155
Sucrose	100	60
Soy oil	40	28
Cellulose	50	50
L-Cysteine	1,8	3,5
Mineral mix*	35	35
Vitamin mix*	10	10
Bitarate choline	2.5	2.5
Lard	-	21.5
BHT**	0.016	-
**Total**	**1000**	**1000**
**Macronutrient (%)**		
Protein	14.7	14.1
Carbohydrate	75.8	40.4
Lipids	9.47	45.4

* Vitamin and Mineral Mix: vit. A, vit. D3, vit., K3, vit. B Complex, Pantothenic Acid, Folic Acid, Biotin, Choline, Vit. C, selenium, iron, copper, manganese, iodine, zinc, cobalt, calcium and phosphorus.

** butylated hydroxytoluene.

The experimental protocol (schematized in Figure 1) lasted 27 weeks and was divided into two phases: (i) an obesity induction and maintenance phase of 19 weeks, followed by (ii) an 8-week treatment period with Cap. At the end of the 19th week, rats were stratified according to body weight, randomly assigned, and distributed into three groups based on the presence or absence of capsinoid treatment: Control (C, n = 10), Obese (Ob, n = 12), and Obese plus capsinoids (ObCap, n = 13).

**Figure 1:** Schematic representation of experimental protocol (27 weeks). "SD": standard diet (n = 16); "HFD": high-fat diet (n = 25); Control (C, n= 10), Obese (Ob, n= 12) and Obese plus capsinoids (ObCap, n= 13).

### Administration of Capsinoids

The ObCap group received capsinoids (CAP; Infinity Pharma, Brazil) daily via orogastric gavage at a dosage of 10 mg of Cap per kg of body weight, diluted in 1 mL of water per kg of body weight, for an 8-week period.^21^ The Cap dose was recalculated weekly to account for changes in body weight, ensuring consistent administration throughout the study. The C and Ob groups received vehicle gavage in equivalent volumes.

### Nutritional assessment

Nutritional assessment was performed using weekly body weight measurements, the sum of epididymal and visceral fat, and calculation of the adiposity index (total fat/final body weight × 100).^22,23^

### Biochemical and hormonal profiles

The animals’ glycemic profiles were assessed after a 6-hour fast in the final week of the protocol. Glycemic levels were measured at baseline and at 30, 60, 90, and 120 minutes following an intraperitoneal injection of 50% glucose solution.^23^ Glucose tolerance was evaluated by calculating the area under the curve (AUC) for glucose. Insulin resistance was analyzed using the HOMA-IR index (homeostatic model assessment), calculated as [fasting insulin concentration (μU/mL) × fasting blood glucose (mmol/L)/22.5], based on plasma glucose and fasting insulin concentrations.

Hormonal profiles (leptin and insulin) were determined using specific kits (Anti-Insulin Merck^®^ EZRMI-13K). The lipid profile was assessed by measuring plasma triglycerides (TG) and total cholesterol levels with specific kits (Bioclin Bioquímica, Brazil).

### Cardiac remodeling assessment – Macroscopic Analysis

Macroscopic remodeling, indicative of myocardial hypertrophy, was assessed by total heart mass and normalized by tibial length.

### Histological Analysis

The transverse sectional area of the left ventricle from animals in each group was fixed in 4% paraformaldehyde and embedded in paraffin. Sections of 6 μm were obtained and stained with hematoxylin-eosin (HE) to determine the myocyte cross-sectional area. All analyses were performed blind.

### Isolation of cardiac myocytes

The cardiac functional analysis *in vitro* was performed using the isolated cardiomyocyte technique.^18^ Cellular contractility was assessed using the cardiomyocyte length variation technique with an inverted microscope with an edge detection system. Cells, maintained in Tyrode's solution and stimulated at 1Hz, had their contractile responses recorded and analyzed for percentage shortening, maximum shortening and relaxation rates, and the time to 50% of peak shortening and relaxation.^21^ All analyses were performed blind.

### Statistical analysis

Data distribution was assessed using the Shapiro-Wilk normality test. Results are presented as mean ± standard deviation (SD). Comparisons between SD and HFD were conducted using Student's t-test for independent samples. For comparison between C, Ob, and Obcap groups one-way ANOVA followed by Tukey's *post-hoc* test was used. To assess changes in body weight over the period of exposure to the experimental diets, two-way ANOVA was used, followed by Bonferroni's *post hoc* test. During the Cap treatment period, body mass and GTT assessments were performed using two-way ANOVA, with Bonferroni's post hoc test for subsequent analysis. To assess contractility parameters, Kruskal-Wallis was used, followed by Dunn's *post hoc* test. A significance level of 5% was set for all tests. Statistical analyses and graphical representations were performed using GraphPad Prism 9.0 software.

## Results

### Characterization of obesity

Initially, the animals exhibited similar body weights during the first 15 weeks of the experimental protocol (Figure 2). From the 16th week onward, however, the HFD group (465 ± 143 g) showed a significantly higher body weight compared to the SD group (438 ± 128 g), marking the onset of obesity. The period between the 16th and 19th weeks was designated as the obesity maintenance phase, during which the groups were reclassified as Control (C) and Obesity (Ob).

**Figure 2:** Evolution of body weight in the standard diet group (SD, n = 16), fed a normocaloric diet, and in the high-fat diet group (HFD, n = 25), fed a high-fat diet during the obesity-induction period. After the onset and characterization of obesity, the groups were reclassified as Control (C, n = 16) and Obese (Ob, n = 25). Values are expressed as mean ± SD. Two-way ANOVA followed by Bonferroni post hoc test. Data were considered significant when p < 0.05 for Ob vs. C.

### Chronic administration of capsinoids

Considering the nutritional profile, during chronic Cap administration a significant reduction in food consumption was observed in the Ob group (15.2 ± 1.7 g/day) compared to the C group (17.6 ± 1.5 g/day). However, caloric intake and feed efficiency did not differ significantly between groups (data not shown). Thus, Cap treatment did not promote improvements in the nutritional profile under obesity conditions.

Table 2 presents the cardiometabolic risk factors based on biometric parameters of the groups after 8 weeks of chronic Cap administration. Regarding body weight, the initial body weight (IBW) of the Ob group was higher compared to the C group.

**Table 2:** Nutritional profile and body composition after treatment with Cap

Variables	Groups		
	C (n=10)	Ob (n=12)	ObCap (n=13)
Food consumption (g/day)	17.6 ± 1.5	15.2 ± 1.7*	16.5 ± 7.3
Caloric intake (Kcal/day)	67 ± 6	73 ± 8	70 ± 10
Feed efficiency (%)	0.3 ± 1.4	0.7 ± 1.2	0.4 ± 0.9
Initial body mass (g)	539 ± 50	593 ± 53*	600 ± 48
Final body mass (g)	541 ± 45	583 ± 54	608 ± 44
Epididimal fat (g)	8.3 ± 1.9	1.6 ± 1.9**	13.0 ± 2.2
Visceral fat (g)	7.0 ± 1.6	11.7 ± 2.4***	13.0 ± 2.7
Sum of fat pads (g)	28.6 ± 3.5	43.6 ± 8.1**	52.4 ± 12.8
Adiposity index (%)	5.3 ± 0.8	7.5 ± 1.1**	8.6 ± 1.9

Data are shown as mean ± SD. Control (C), Obese (Ob) and Obese plus capsinoids (ObCap). One-way Anova followed by Tukey's pos hoc test.

* p<0,05,

** p<0,01,

*** p < 0.001;

**** – vs. C.

As expected, rats in the Ob group exhibited elevated values for all adiposity parameters compared to the C group, confirming the effectiveness of the high-fat diet in inducing obesity. However, Cap treatment did not reduce or prevent the fat accumulation associated with obesity (Table 2).

Figure 3 illustrates obesity-related comorbidities in the groups following chronic Cap treatment. At baseline, the ObCap group showed elevated glycemic levels compared to the Ob group. No significant differences were observed at other time points (Figure 3A). Additionally, no differences were found in the area under the curve (AUC) for glucose or in triglyceride levels between groups (Figure 3B and 3G).

**Figure 3:** Lipid, glycemic and hormonal profiles after treatment with Cap. A) Glucose Tolerance Test, ** p < 0.01 ObCap vs. Ob; Control (C, n= 6), Obese (Ob, n= 7) and Obese plus capsinoids (ObCap, n= 10). B) Area under the curve (AUC) for glucose; Control (C, n= 6), Obese (Ob, n= 7) and Obese plus capsinoids (ObCap, n= 10). C) Insulin; Control (C, n= 5), Obese (Ob, n= 7) and Obese plus capsinoids (ObCap, n= 5). D) HOMA-IR (Homeostatic Model Assessment - Insulin Resistance); Control (C, n= 5), Obese (Ob, n= 6) and Obese plus capsinoids (ObCap, n= 5). E) HOMA-β (Homeostatic Model Assessment - Beta); Control (C, n= 5), Obese (Ob, n= 6) and Obese plus capsinoids (ObCap, n= 5). E) Leptin; Control (C, n= 7), Obese (Ob, n= 7) and Obese plus capsinoids (ObCap, n=v7). G) Triglycerides; Control (C, n= 10), Obese (Ob, n= 10) and Obese plus capsinoids (ObCap, n= 10) and; H) Cholesterol; Control (C, n= 9), Obese (Ob, n= 9) and Obese plus capsinoids (ObCap, n= 9). Values are expressed as mean ± SD and individual values. Statistical comparisons were performed using two-way ANOVA followed by Bonferroni's test for multiple comparison (A) or by one-way ANOVA followed by Tukey's test for multiple comparison (B - H). Data were considered significant when * p < 0.05; ** p < 0.01; *** p < 0.001.

Insulin levels were significantly elevated in the Ob group compared to the C group, but there was no significant difference between the Ob and ObCap groups (Figure 3C). A similar pattern was observed for the HOMA-IR index, with the Ob group showing a higher index compared to the C group. For the HOMA-β index, the Ob group showed an increase compared to the C group (Figure 3D and E). It is important to note that there were no differences for these indices between Ob and ObCap, suggesting no effect of Cap treatment.

Regarding leptin levels, the Ob group showed a significant increase compared to the C group, with no significant difference between the Ob and ObCap groups (Figure 3F). The Ob group had higher cholesterol levels compared to the C group (Figure 3H).

Regarding cardiac morphology, the results showed that the Ob group showed an elevation in heart weight compared to the C group (Figure 4A); however, when heart weight was normalized by tibia length, there were no significant differences between groups (Figure 4B). Cellular morphometry was assessed by examining sarcomere length and cardiomyocyte cross-sectional area. Neither Cap treatment nor obesity were able to induce changes in these parameters (Figure 4C-E).

**Figure 4:** Cardiac morphological characteristics after treatment with Cap. A) Heart weight; Control (C, n= 5), Obese (Ob, n= 7) and Obese plus capsinoids (ObCap, n= 7). B) Heart/tibia length; Control (C, n= 5), Obese (Ob, n= 7) and Obese plus capsinoids (ObCap, n= 7). C) Sarcomere length; Control (C, n= 5), Obese (Ob, n= 5) and Obese plus capsinoids (ObCap, n= 5). D) Representative images of left ventricular transverse sections (×40) stained by H&E, scale bar= 50 µm. E) Bars graph of cross-sectional area (CSA); Obese (Ob, n= 7) and Obese plus capsinoids (ObCap, n= 7). Values are expressed as mean ± SD and individual values. Statistical comparisons were performed using one-way ANOVA followed by Tukey's test for multiple comparison (A – C, E). Data were considered significant when * p < 0.05 vs. C.

To better understand the cellular effects of Cap treatment in the presence of obesity, experiments with isolated cardiomyocytes were performed to analyze the contractile function of the cells. Cap treatment surprisingly elicited a significant modulation in cardiomyocyte contractile, as evidenced by a reduction in fractional shortening in the ObCap group compared to Ob (Figure 5A). Parameters such as the maximum shortening and relaxation rates remained unchanged with both Ob and ObCap groups (Figure 5B-C). Ob decreased the time to 50% shortening compared to the C group, whereas the ObCap group showed an increase in this parameter relative to the Ob group (Figure 5D). Time to 50% relaxation was not different among groups, (Figure 5E).

**Figure 5:** Effect of Capsinoids on the functional parameters of isolated cardiomyocytes. A) Fractional shortening expressed as % of resting cell length; Control (C, n=65 cells), Obese (Ob, n= 61 cells) and Obese plus capsinoids (ObCap, n= 60 cells). B) Maximum rate of shortening; Control (C, n=65 cells), Obese (Ob, n= 61 cells) and Obese plus capsinoids (ObCap, n= 60 cells). C) Maximum rate of relaxation; Control (C, n=65 cells), Obese (Ob, n= 60 cells) and Obese plus capsinoids (ObCap, n= 60 cells). D) Time to 50% shortening; Control (C, n=64 cells), Obese (Ob, n= 60 cells) and Obese plus capsinoids (ObCap, n= 60 cells). E) Time to 50% relaxation; Control (C, n=65 cells), Obese (Ob, n= 61 cells) and Obese plus capsinoids (ObCap, n= 59 cells). Data are expressed as median ± interquartile ranges. Statistical comparisons were performed using Kriskal-Wallis followed by Dunn's test for multiple comparison (A – E). Data were considered significant when * p < 0.05; ** p < 0.01; *** p < 0.001.

## Discussion

The main results demonstrated that chronic administration of Cap, as a pharmacological approach to obesity treatment, was not effective in reducing adiposity. Moreover, chronic Cap administration did not improve glycemic, lipid, or hormonal profiles and failed to prevent isolated hypercholesterolemia and hyperinsulinemia associated with obesity. However, specifically in the heart, Cap treatment compromised cellular contractility, as evidenced by reduced fractional shortening and increased time to 50% shortening, highlighting its ability to modulate cardiomyocyte contractility.

Various pharmacological interventions have been employed to treat obesity,^24,25^ and among these approaches, Cap has been investigated as a non-pharmacological tool with promising effects in reducing body mass and adiposity in obese mice.^26,27^

The results indicate that from the 16th week onward, the Ob groups exhibited statistically significant differences in body weight, maintaining this difference until the end of the obesity induction and maintenance protocol (19th week). In the context of the present study, the data show that the intervention with Cap was not effective in reducing body parameters or adiposity. Overall, our results demonstrate that chronic Cap treatment had no impact on body fat accumulation, revealing a previously little-explored interaction. This finding is noteworthy because Cap has emerged as a compound with potential actions for controlling and reducing obesity, given its reported ability to increase the expression of proteins related to lipid metabolism, enhance oxygen consumption, and reduce body mass and fat deposits.^28^

A possible explanation for the lack of effect of Cap on body weight and adiposity may be related to the dose used. In addition, methodological differences, such as varied diets (with or without lard) and different treatment durations, can lead to varied results in the short term, but hich may not be sustained in the long term.^24,29,30^ Another relevant point is that some studies that reported positive results with capsaicin and *Capsicum annum* used the substance mixed in the feed, which can influence the total amount ingested by each animal.^25,30^

Although the available literature on the mechanism of action of Cap treatment is limited, this study did not directly evaluate the expression, activity or modulation of possible mechanistic targets. However, evidence relating to its analogues is relevant, as some of these can produce effects similar to capsaicinoids, such as promoting thermogenesis, stimulating energy and lipid metabolism, and reducing body mass and adipose tissue.^17,18^ Furthermore, these compounds can improve glucose tolerance, exhibit anti-hyperlipidaemic activity and demonstrate anti-inflammatory and antioxidant properties.^8,21^

Capsaicin stands out in this scenario, as it acts by activating TRPV1 and can exert beneficial effects in the context of obesity.^20^ These mechanisms were considered in the discussion as possible explanations for the observed effects. However, important gaps remain regarding the specific action of Cap and the possibility that its effects are similar to those described for capsaicin. Thus, further targeted studies are needed to investigate the role of Cap and the mechanisms involved in its action.

In this context, the study by Kang et al.,^20^ which used a high-fat diet-induced obesity model in mice, demonstrated that dietary capsaicin can reduce obesity-induced glucose intolerance *in vivo*. In addition, the benefits of capsaicin are associated with the reduction of inflammatory phenotypes in adipose tissue and the liver, two peripheral tissues that play crucial roles in insulin responsiveness. These findings suggest that dietary capsaicin may counteract the development of obesity-associated glucose intolerance and potentially insulin resistance. According to Kwon et al.,^26^ this effect may be attributed to TRPV1 activation in the pancreas, which can induce the release of calcitonin gene-related peptide (CGRP) and stimulate insulin secretion. Moreover, TRPV1 agonists can increase intracellular Ca^2+^ levels in β-cells, expand β-cell mass, and promote greater insulin secretion. However, to better understand the mechanisms underlying these effects, further studies specifically investigating the action of Cap are needed.

In contrast to this idea, our data clearly show that chronic administration of Cap failed to prevent obesity-induced hyperinsulinemia and insulin resistance. Our findings are supported by previous evidence showing that Cap treatment did not improve insulin levels, insulin resistance, or leptin concentrations in obese rats.^21^

Similarly, our findings show that treatment with Cap was not effective in reversing isolated hypercholesterolemia. In this context, Cansian^8^ also investigated the effects of the dry extract of Capsicum annuum L (40%) on the lipid profile of obese rats treated for 6 weeks. However, no changes were observed in serum levels of total cholesterol, HDL and triglycerides. However, a study with enzymatically synthesized capsiate and dihydrocapsiate found that treatment resulted in a reduction in serum cholesterol levels and serum lipids.^31^

The association between diet and increased body fat has been linked to the emergence of various comorbidities, increasing the risk of developing cardiovascular diseases.^32^ The literature frequently reports that obesity promotes cardiac remodeling, which is characterized by morphological, molecular and functional alterations resulting from changes in cardiac volume, mass and function in response to pathophysiological aggressions.^2,33^

Our results showed no changes in cardiac morphology between the different groups of the experiment. The increase in absolute heart weight observed in obese animals did not persist after normalization of tibia length, indicating that cardiac growth occurred proportionally to the increase in body weight. Furthermore, the analyses did not show an increase in cross-sectional area (CSA) or sarcomere length, suggesting that the observed changes reflect a physiological adaptation to increased body mass, without evidence of pathological cardiac hypertrophy.

In the current study, we demonstrated for the first time that chronic Cap treatment reduced cardiomyocyte contractile in the obesity. This discovery is important because there is little information available on the effects on the heart and cellular contractility of Cap treatment in the context of obesity. Studies investigating cardiac contractile function in experimental models of obesity reveal a deterioration in contraction capacity, evidenced by a decrease in the percentage and speed of cardiomyocyte shortening.^24,34^

Our results show that the shortening fraction of cardiomyocytes from obese animals is preserved, with a reduction in the time required to reach 50% of maximum shortening. This finding may be associated with alterations in intracellular calcium handling or changes in contractile protein sensitivity; however, the underlying mechanisms have not yet been directly evaluated. Therefore, despite the preservation of contractile function, the change in contraction speed suggests modifications in cardiomyocyte mechanics related to obesity. Although the literature consistently reports impairments in myocardial contractility associated with obesity, our results do not demonstrate such dysfunction. This discrepancy may be explained by variations in the experimental model, such as species, strain, or dietary protocol, which could influence cardiomyocyte susceptibility to functional alterations.^33^ It is also possible that cellular adaptations occur, such as increased myofibrillar sensitivity to calcium.^35^

When we compared cardiomyocytes from obese animals treated with Cap to those from the untreated obese group, we observed a reduction in the shortening fraction and an increase in the time required to reach 50% of maximum shortening (see central illustration). These results indicate impaired cellular contractile function in terms of both magnitude and speed. While the obese group maintained contraction amplitude, Cap treatment reduced shortening capacity and slowed contraction, suggesting diminished mechanical efficiency. This effect may be related to alterations in intracellular calcium handling, possibly through modulation of regulatory proteins such as SERCA2a and RYR2, which could reduce calcium availability for contraction.^35^ Further studies are needed to elucidate the mechanisms underlying the action of Cap in the cardiac system. A limitation of this study is the absence of an evaluation of cardiac function *in vivo*, which restricts our understanding of the extent to which the observed cellular changes translate into organ-level functional outcomes.

Although there is no literature describing the mechanism by which Cap treatment affects cardiomyocyte contractility, evidence from its analogue capsaicin provides relevant context. Capsaicin is known to act on the cardiac system through the TRPV1 receptor, exerting cardioprotective effects by reducing infarct size and stimulating vasodilation.^36^ However, significant gaps remain in our understanding of how Cap acts on the cardiac system and whether its effects resemble those attributed to capsaicin. Therefore, further studies are needed to investigate the role of Cap in cardiac function and contractility, as well as the mechanisms involved.

In this context, capsaicin treatment has been shown to improve cardiovascular autonomic control in newborn rats, restoring heart rate. At high doses, capsaicin was also able to increase sympathetic modulation of vascular tone.^37^ Studies have demonstrated that the acute effect of capsaicin in cardiomyocyte cultures increases intracellular Ca^2+^ concentration and attenuates fractional shortening through TRPV1 stimulation, as evidenced by the use of a specific TRPV1 blocker that prevented the capsaicin-induced rise in intracellular Ca^2+^.^38,39^

More recently, Isaev et al.^40^ investigated the effects of capsaicin on voltage-dependent ionic currents in rabbit ventricular myocytes. Capsaicin inhibited rapidly activated (IKr) and slowly activated (IKs) K+ currents, as well as the transient outward K+ current (Ito). At higher concentrations, capsaicin also suppressed voltage-gated Na^+^ and Ca^2+^ currents and the inward rectifier IK1. Overall, these findings suggest that capsaicin influences cardiac electrophysiology by interacting with multiple ion channels. Therefore, caution is advised when administering capsaicin to individuals with cardiac channelopathies or those prone to arrhythmias, such as patients with ischemic heart disease.

Taken together, our results provide evidence that Cap induces impaired cardiomyocyte contractility in the context of obesity. These findings suggest that, despite preserved cardiac morphology, Cap treatment may negatively affect the cellular mechanics of cardiac myocytes.

## Conclusion

Treatment with Cap modulates cardiomyocyte contractility in the presence of obesity. Our findings provide new insights into the relationship between Cap treatment and cardiomyocyte contraction, but the therapeutic potential needs further clarification. Furthermore, Cap treatment did not improve the fat accumulation phenotype caused by obesity induction, nor did it improve metabolic parameters.

## Data Availability

The underlying content of the research text is contained within the manuscript.
